# Synthesis of Au/SnO_2_ nanostructures allowing process variable control

**DOI:** 10.1038/s41598-019-57222-z

**Published:** 2020-01-15

**Authors:** Myung Sik Choi, Han Gil Na, Sangwoo Kim, Jae Hoon Bang, Wansik Oum, Sun-Woo Choi, Sang Sub Kim, Kyu Hyoung Lee, Hyoun Woo Kim, Changhyun Jin

**Affiliations:** 1 Division of Materials Science and Engineering, Hanyang University, Seoul, 04763 Republic of Korea; 2 Liquid Processing and Casting R&D Group, Korea Institute of Industrial Technology, 156, Getpearl-ro, Yeonsu-gu, Incheon, 21999 Republic of Korea; 3 Department of Materials Science and Engineering, Kangwon National University, Samcheok, 25913 Republic of Korea; 4 Department of Materials Science and Engineering, Inha University, Incheon, 402-751 Republic of Korea; 5 Department of Materials Science and Engineering, Yonsei University, Seoul, 03722 Republic of Korea

**Keywords:** Materials chemistry, Chemical engineering

## Abstract

Theoretical advances in science are inherently time-consuming to realise in engineering, since their practical application is hindered by the inability to follow the theoretical essence. Herein, we propose a new method to freely control the time, cost, and process variables in the fabrication of a hybrid featuring Au nanoparticles on a pre-formed SnO_2_ nanostructure. The above advantages, which were divided into six categories, are proven to be superior to those achieved elsewhere, and the obtained results are found to be applicable to the synthesis and functionalisation of other nanostructures. Furthermore, the reduction of the time-gap between science and engineering is expected to promote the practical applications of numerous scientific theories.

## Introduction

Currently, although the boundaries of the academic area do not seem to be important, a clear-cut borderline separates pure science^1,2^, which explores the principles of nature, from applied engineering^3,4^, which deals with real-life processes. This separation largely reflects the corresponding difference in the utilised approaches, highlighting the fact that the application of new theories to real-world problems is difficult and time-consuming. For this reason, scientific heritage newly published every day is often discarded without actually being phenomenologically expressed. On the contrary, our daily life presents numerous strange phenomena that cannot be scientifically explained because of the lack of a proper academic background. That is, there may be cases of a theory not backed by experimental results or results not explained by any theory. As mentioned above, science and engineering can be viewed to be in a state of temporal hysteresis, and the search for ways of narrowing the corresponding time gap should therefore be regarded as a task of high significance. For example, Shi *et al*. reported the hetero-structured AgBr/ZnO photocatalyst, but their synthesis requires long reaction times and complex multi-step processes^5^. Ellis *et al*. also proposed the morphology control of hydrothermally prepared LiFePO4 with long reaction times and post heat treatment processes^6^. In other words, engineering techniques that can easily and economically confirm competitive scientific theories are shortcuts that can reduce the time cost of the practical application of science and achieve unique and meaningful results. Unluckily, because of the atmosphere that emphasises originality in research, one tends to think that only complicated and difficult-to-perform experiments can produce unique results. However, the reason why we cannot conclude that it is preconceived is that many of the results have received good evaluation in the meantime. For example, when studies on various nanostructures^7–10^ performed so far are divided into those dealing with morphology^11,12^, crystallography^13,14^, and elemental composition^15,16^ control, one can recognise that these investigations have a certain research value when the desired shape, microstructure, or function has been fully achieved. In this case, the employed raw materials and equipment are costly, the use of in-house-made equipment precludes verification in other laboratories, the experiment condition that was different from the existing experiment was exactly met in the repeated experiment, and technological differences related to the use of high-end analytical equipment are rarely considered. In other words, the outcomes of such experiments emphasise specificity rather than generality, and consequently require much time to be verified by engineering in real life, i.e., in such cases, one can only imply that a new theory can be realised. Herein, we introduce new processing advantages to easily fabricate a heterogeneous structure by attaching Au particles to pre-formed SnO_2_ nanostructures and compare the advantages of our work with the disadvantages of existing works in six representative categories used in science/engineering fields. The proposed method allows one to induce nucleation and growth in nanostructures in a shorter time than in the case of other synthesis/deposition techniques. Moreover, thermal energy injection allows the phase change and composition to be relatively easily altered, and the developed technique also allows one to easily change the shape and microstructure of pre-formed nanostructures, which is attributed to energy injection variation with temperature and holding time. Thus, in contrast to the existing principle of one-to-one matching, which assumes that one factor depends on one process variable, the described technique utilises a new one-to-many matching concept, allowing one to simultaneously control multiple factors with one process variable.

## Result and Discussion

**Figure 1:**
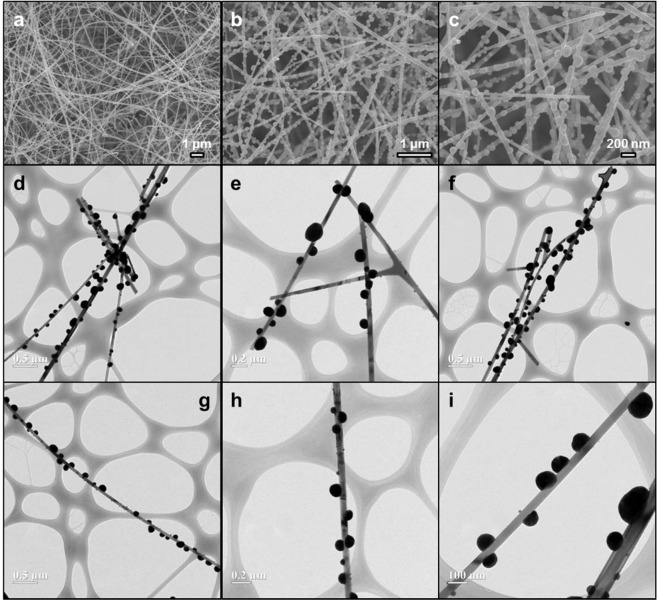
Typical SEM and TEM images of SnO_2_ nanowires formed by conventional thermal evaporation and of Au particles formed on SnO_2_ nanowires by flame chemical vapour deposition. (**a**) SEM image of bare SnO_2_ nanowires with a smooth surface; (**b**) low-magnification and (**c**) high-magnification SEM images of Au-SnO_2_ hybrid nanostructures; (**d–i**) variable-magnification TEM images of Au-SnO_2_ nanostructures of different shapes.

Figure 1 shows Au particle–decorated SnO_2_ nanostructures prepared under various experimental conditions. As is well known^17,18^, smooth and long SnO_2_ nanowires can be easily synthesised by thermal evaporation of Sn powder in an oxygen-containing atmosphere. Herein, the thickness of SnO_2_ nanowires ranged from 20 to 120 nm, and their length ranged from several tens to several hundred μm (Fig. 1a–c). However, when a 5-s energy pulse was applied to SnO_2_ nanowires that had previously been exposed to HAuCl_4_⋅4H_2_O/(CH_3_)_2_CHOH, the originally smooth surface of SnO_2_ nanowires got covered by Au particles and therefore became rough. The spherical Au particles attached to nanowires had a size of roughly 100–300 nm and were in a discrete state (Fig. 1d–i). Specifically, these particles did not aggregate to lower their surface energies and existed independently at regular intervals, which was ascribed to the fact that the thermal energy applied to SnO_2_ nanowires was not concentrated in a narrow region adjacent to nanowires but was uniformly dispersed in space. In other words, it was concluded that the applied thermal energy allowed the rate of nucleation to be held constant at all points of SnO_2_ nanowires. Thus, it could be said that this simultaneous energy injection was different from the general mechanism of nucleation and growth in the local region of interest. Only morphologically, Au-decorated SnO_2_ nanostructures can be prepared in any number of ways. However, most of these methods refrain from instantaneous processing to increase the cross-sectional area and require various pre- and post-processing techniques^19–21^. For example, Kim *et al*. synthesised noble metal–decorated SnO_2_ nanowires using thermal activation and employed the large cross-sectional area of these nanoparticles to detect noxious gases^22^. Wu *et al*. suggested that hydrothermally prepared hollow hybrid Au-SnO_2_ nanostructures can be utilised in photocatalysis application^23^, with advantages such as environmentally friendly solution-basis, low-cost, and surfactant-free. Bing *et al*. used a rational combinational multi-step synthetic route to prepare Au-loaded SnO_2_ hollow multi-layered nanosheets, composed of numerous nanoparticles as structural subunits^24^. Furthermore, although previous reports could realise nanocomposite structures of the abovementioned morphology^25,26^, they remain inferior in terms of speed, accuracy, yield, and economy, i.e., accessibility. For example, Lai *et al*. realised heterogeneous nucleation sites using a template with a low vaporisation point and fabricated nanobeads by subsequent template removal at high temperatures^27^. Some studies suggested that Pd-functionalised nanostructures can be formed by post-heat treatment and/or electron beam irradiation, without the involvement of extra precursors, because extra energy itself facilitates nucleation at an energy lower than that of homogeneous nucleation^28,29^. However, in the two cases mentioned above, there is no way to control nanowire parameters until the end stage, since metal particle nuclei are formed on the metal oxide before or during synthesis. That is, if the desired metal-decorated nanowires are not obtained, the experiment needs to be re-started from the very beginning. In contrast, our method relies on simultaneous annealing, allowing one to rapidly functionalise existing SnO_2_ nanowires in the desired way. This advantage cannot be found in any other post-processing technique, and one can therefore say that in addition to the abovementioned five advantages (speed, accuracy, yield, economy, and accessibility), our method also guarantees stability.

As shown in Fig. 1d–i, even though rugged Au particles were commonly observed on the originally smooth SnO_2_ nanowires, bigger Au spheres were sometimes formed on the surface of certain SnO_2_ nanowires through interfacially controlled spherical growth^30^. The above figures indicate no change in the morphology of Au-decorated nanowires; however, even in a localised region, the size of Au particles produced on nanowires tended to decrease with increasing time of thermal energy injection in that region. This behaviour was ascribed to the role of injected heat energy in inducing simultaneous nucleation over a large area, so that the effect of growing the nucleated first in a momentary difference is relatively insufficient. Therefore, as the retention time of energy injection at each point increased, the distance between Au particles generated in the nanowire decreased, while the density of Au particles produced at a fixed length increased. These results are in stark contrast with the fact that after nucleation, the rapid growth of nanostructures is commonly controlled by the rate of constituent atom diffusion^31^.

**Figure 2:**
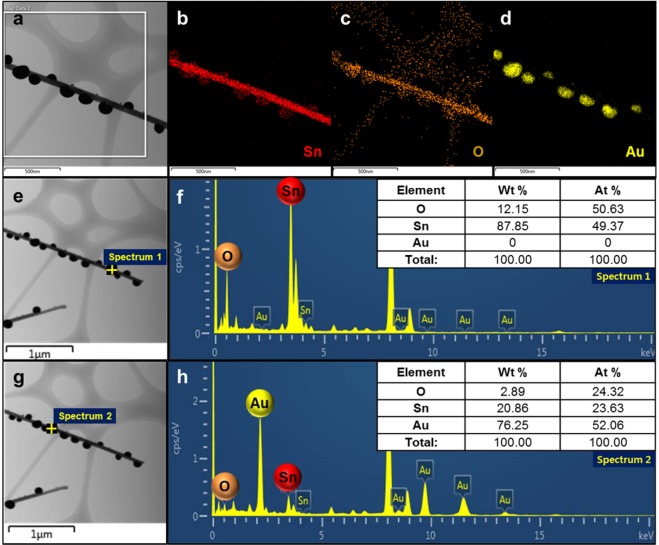
Zone composition of Au-SnO_2_ hybrid nanostructures identified by elemental mapping and EDX. (**a**–**d**) Distributions of Sn, O, and Au in a typical Au-decorated SnO_2_ nanowire; (**e**,**f**) contents of Sn and O in a SnO_2_ nanowire determined excluding Au particles; (**g**,**h**) contents of Sn, O, and Au in a Au particle excluding the SnO_2_ nanowire.

The above finding indicates that the increase of supplied energy amount with increasing heating time is related to the reduction of SnO_2_ accompanied by the formation of Au (Fig. 2). Thus, even for Au-decorated SnO_2_ of the same type, the reduction gradient is determined by the energy injection time (heating time). To investigate the degree of reduction, all samples were subjected to elemental mapping (Fig. 2a–d) and EDX (Fig. 2e–h) measurements, which revealed that the Sn:O ratio was different in nanowires and Au particles, as described above. In SnO_2_ nanowires, Au was not observed at all (Fig. 2e,f), but Sn and O were detected together with Au in the particle region (Fig. 2g,h). That is, the precipitation of Au and the reduction of SnO_2_ occurred simultaneously. This behaviour probably reflects the fact that when the Au solution was pushed to one side to become a particle as a result of energy supply, a part of the SnO_2_ nanowire surface was exposed and lost O because of the effect of direct energy injection. Thus, one has only started exploring the applications of these two effects, which are believed to have much scientific and engineering potential.

**Figure 3:**
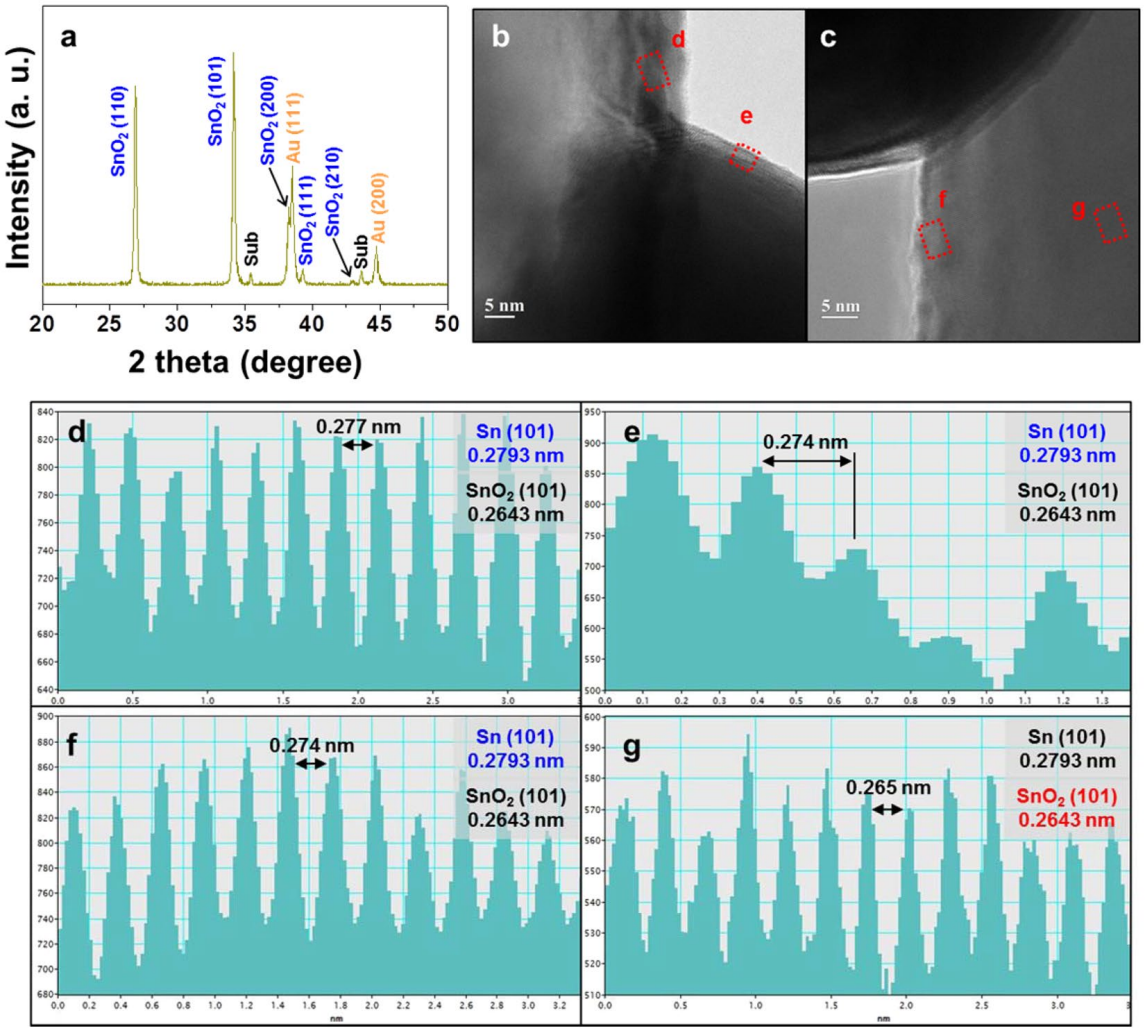
Crystallinity and microstructure of several parts of Au-decorated SnO_2_ nanostructures. (**a**) XRD spectrum of Au-SnO_2_ mixture; (**b**,**c**) HRTEM images acquired at the interface between a Au particle (dark region) and a SnO_2_ nanowire (white region); (**d**) interplanar spacing showing the reduction of SnO_2_ to Sn on the surface of SnO_2_ nanowire; (**e**) interplanar spacing of SnO_2_-based layers formed on the Au particle surface; (**f**) interplanar spacing confirming the reduction of SnO_2_ to Sn, measured on the other surface of the SnO_2_ nanowire; (**g**) interplanar spacing confirming the reduction of SnO_2_ to Sn, measured inside the SnO_2_ nanowire.

For each sample, the crystal phase composition and microstructure were verified by XRD (Fig. 3a) and TEM (Fig. 3b–g), respectively. Bare SnO_2_ nanowires were shown to have a tetragonal structure^32^, and peaks at 2*θ* = 26.61°, 33.89°, 37.95°, 38.97°, and 42.63° were in good agreement with reflections from (110), (101), (200), (111), and (210) planes of tetragonal SnO_2_, respectively (JCPDS No. 41–1445) (Fig. 3a)^33^. Peaks at 2*θ* = 38.18° and 44.39° were ascribed to reflections from the (111) and (200) planes of Au (JCPDS No. 04–0784), respectively^34^ (Fig. 3a). On the other hand, for samples prepared by applying thermal energy to bare SnO_2_ nanowires, we observed a change of SnO_2_ peak intensity and the appearance of new peaks. As described above, a reduction of SnO_2_ to Sn may occur under the employed conditions, which may result in the formation of non-equilibrium SnO_*x*_ (0 < *x* < 2) phases (Fig. 3b–g). Although these peaks did not exactly match those in JCPDS cards because of the non-equilibrium nature of the former, such chemical changes could be sufficiently inferred from the shift of the (101) peak of pre-formed SnO_2_ to the (101) peak of Sn. At this time, a SnO_2_ layer was detected on the surface of SnO_2_ nanowires (Fig. 3d,f) and on the surface of Au particles (Fig. 3e). However, interplanar spacings of unbalanced compositions could be observed with proceeding partial reduction. In other words, no crystal phases except for those of SnO_2_ and Au were observed by XRD, although HRTEM line profiling indicated that the surface profiles of SnO_2_ or Au could change. This means that the energy supplied to the existing SnO_2_ nanowires was sufficient for SnO_*x*_ to form on the Au surface (Fig. 3d–g).

The efficiency of making Au-SnO_2_ should be further objectified to allow the clear application of the competitiveness of the new method and its difference from the existing techniques. Consequently, the process methods were evaluated using six parameters (Tables 1, 2, S1 and S2), namely the employed precursor, equipment, pre- and post-treatment, temperature, time, and vacuum^35–66^. First, the new processing technique was found to be applicable to all materials regardless of their type. Second, while the previously used equipment occupies much space, requiring additional equipment to achieve the special purpose, our processing technology is not affected by location and does not involve the utilisation of useless accessories. Third, researches conducted so far, especially those requiring pre-processing such as templating and post-processing such as heat treatment, have often involved supplementary procedures to address the difficulty of direct synthesis and deposition, whereas our high-efficiency method does not require any pre- or post-processing. Fourth, in previous methods, the temperature had to be maintained within the range of at least 500 to 1000 °C for a long time to adjust the synthesis temperature of heterogeneous materials, whereas our method instantaneously provides a temperature of 1300 °C. Fifth, our process is operated on a timescale of seconds and is clearly different from other processes operated on the time scales of minutes or even hours, allowing one to control instantaneous processing conditions on the spot to match material properties. Sixth, conventional synthesis and deposition equipment requires the use of variable (low to ultra-high) vacuum depending on the specific case, whereas our technique does not require additional vacuum conditions, since it can be operated under atmospheric pressure. There may be many other classification criteria, but it seems clear that the above six advantages provide overwhelming evidence of the superiority of our method.

**Table 1:** Comparison of our process with previously reported ones.

This work	Other work				
	Composites	Precursor	Equipment	Pre- and post- treatment	Ref.
Composites: SnO_2_ NWs - Au NPs	TiO_2_ NTs- Ag NPs	AgNO_3_, Salicylic acid	DC current power supply, stirrer, furnace	anodization, stirring	^35^
	WO_3_ NFs-Rh_2_O_3_ NPs	Rhodium chloride hydrate, butanediol, PVP, sodium citrate, acetone, ammonium metatungstate hydrate	centrifugal separator, stirrer, furnace, DC voltage supply	centrifugation, stirring	^36^
	ZnO NWs-Cr_2_O_3_ NPs	CrCl_2_	furnace	—	^37^
	SnO_2_ NWs- Ag NPs	Ag filament	furnace	—	^38^
Precursor: Gold Chloride hydrate, 2-propanol	WO_3_ NRs- Pd NPs	PdCl_2_, ethanol	furnace	—	^39^
	SnO_2_ NWs-Cr_2_O_3_ NCs	CrCl_2_	furnace	—	^40^
	RuO_2_ NRs- Ru NPs	—	furnace	thermal reduction	^41^
	TiO_2_ NRs- NiO NPs	NiCl_2_·6H_2_O, 2-methoxyethanol, ammonia	stirrer, furnace	stirring	^42^
	TiO_2_ NWs- Au NPs	HAuCl_4_·3H_2_O, NaOH	furnace	AgNO_3_ test	^43^
	ZnO_2_ NWs-TiO_2_ NPs	TiO_2_ suspension (P25), acetylacetone, Triton X-100, D.I water, ethanol, acetic acid	furnace, sonicator	sonication	^44^
Equipment: FCVD equipment	WO_3_ NWs- PdO NPs	Palladium acetylacetonate, acetone	stirrer, sonicator, furnace	stirring, sonication, furnace	^45^
	WO_3_ NWs- PtO_x_ NPs	Platinum acetylacetonate, acetone	stirrer, sonicator, furnace	stirring, sonication, furnace	^45^
	CuO NWs- Au NPs	Au target	sputter, furnace	sputtering	^46^
	SnO_2_ NWs- Pd NPs	Au target, Sn powder, pluronic (P123) surfactant, PdCl_2_, NaCl_2_	sputter, furnace, stirrer	sputtering, stirring	^47^
Pre- and post- treatment: Nothing	TiO_2_ NFs- Pt NPs	Ethylene glycol, PVP, H_2_PtCl_6_	furnace	—	^48^
	ZnO NWs- Au NPs	HAuCl_4_·4H_2_O, ethanol	UV box, furnace	—	^49^
	ZnO NWs- Pd NPs	PdCl_2_	furnace	—	^50^
	MoO_3_ NWs- Ag NPs	AgNO_3_	stirrer, vacuum oven	stirring, filtering, post-cleaning	^51^
	SnO_2_ NFs- Pt NPs	H_2_PtCl_6_, ethylene glycol, PVP, acetone	centrifugal separator, furnace	centrifugation, post-cleaning	^52^
	ZnO NWs- Au NPs	HAuCl_4_, Na_2_CO_3_	stirrer, furnace	stirring	^53^
	WO_3_ NWs- Pd NPs	PdCl_2_, D.I water, HF	furnace, sonicator	sonication	^54^
	WO_3_ NWs- Pt NPs	Ethylene glycol, PVP, H_2_PtCl_6_, acetone, D.I water, ethanol	furnace, centrifugal separator	centrifugation, post-cleaing	^55^
	SnO_2_ NWs-Cr_2_O_3_ NPs	Cr target	sputter, furnace	sputtering	^56^
	ZnO NWs- CdS NPs	CdSO_4_, NH_4_OH	furnace	—	^57^
	V_2_O_5_@ZnO- Au NPs	HAuCl_4_, D.I water	furnace	—	^58^
	ZnO NW- Au NPs	Citrated-stabilized Au	—	—	^59^
	SnO_2_ NFs- Pt NPs	H_2_PtCl_6_, ethylene glycol, PVP, acetone, D.I water	furnace, centrifugal separator	centrifugation, post-cleaning	^60^
	Zn_2_SnO_4_ NWs-ZnO QDs	Zinc acetate dihydrate, ethanol	autoclave	—	^61^
	GaN NWs- TiO_2_ NCs	TiO_2_ target	RF sputter, furnace	sputtering	^62^
	SnO_2_ NWs- NiO NPs	NiO	furnace	thin film deposition	^63^
	SnO_2_ NWs- CdS QDs	CdSO_4_, thiourea, ammonia	oil bath, furnace	—	^64^
	ZnS NWs- CuO NPs	CuSO_4_, NaOH, D.I water, acetone, isopropyl alcohol	furnace, sonicator, stirrer, centrifugal separator	sonication, stirring, centrifugation	^65^
	TiO_2_ NWs- Ag NPs	D.I water, ethanol, NaOH, AgNO_3_	sonicator, furnace	sonication	^66^

**Table 2:** Comparison of our process with previously reported ones.

This work	Other work				
	Composites	Temp.	Time required	Degree of vacuum	Ref.
	TiO_2_ NTs- Ag NPs	500 °C 80 °C 80 °C 500 °C	2 hr (500 °C) 3 hr (80 °C) 1 hr (80 °C) 3 hr (500 °C)	—	^35^
Composites: SnO_2_ NWs - Au NPs	WO_3_ NFs-Rh_2_O_3_ NPs	686 °C 600 °C	20 min (686 °C) 1 hr (600 °C)	Air	^36^
	ZnO NWs-Cr_2_O_3_ NPs	630 °C	20 min	~9 × 10^−2^ torr	^37^
	SnO_2_ NWs- Ag NPs	300 °C	1 hr	~10^−6^ torr	^38^
	WO_3_ NRs- Pd NPs	500–700 °C	30 min (500–700 °C)	0.1 torr	^39^
	SnO_2_ NWs-Cr_2_O_3_ NCs	620 °C	20 min	~9 × 10^−2^ torr	^40^
Temp.: 1300 °C	RuO_2_ NRs- Ru NPs	650 °C 130 °C	20–120 min (650 °C) 1 hr (130 °C)	~4 × 10^−5^ torr (650 °C) 0.4–1.0 torr (130 °C)	^41^
	TiO_2_ NRs- NiO NPs	40 °C 60 °C 600 °C	1 hr (40 °C) 10 min (60 °C) 1 hr (600 °C)	1 torr	^42^
	TiO_2_ NWs- Au NPs	70 °C 100 °C 200 °C	2 hr (70 °C) 12 hr (100 °C) 4 hr (200 °C)	Air	^43^
	ZnO_2_ NWs-TiO_2_ NPs	450 °C	30 min	Air	^44^
Time required: 5 s	WO_3_ NWs- PdO NPs	300 °C	2 hr	Air	^45^
	WO_3_ NWs- PtO_x_ NPs	300 °C	2 hr	Air	^45^
	CuO NWs- Au NPs	500 °C	30 min	Air	^46^
	SnO_2_ NWs- Pd NPs	45 °C	12 hr	—	^47^
	TiO_2_ NFs-Pt NPs	110 °C	30 min	—	^48^
	ZnO NWs-Au NPs	480 °C	1 hr	—	^49^
	ZnO NWs-Pd NPs	400 °C	4 hr	Air	^50^
Degree of vacuum: Air	MoO_3_ NWs- Ag NPs	0 °C RT (25 °C) 80 °C	30 min (0^o^C) 24 hr (RT) 2 hr (80 °C)	—	^51^
	SnO_2_ NFs-Pt NPs	500 °C	2 hr	Air	^52^
	ZnO NWs-Au NPs	400 °C	4 hr	—	^53^
	WO_3_ NWs-Pd NPs	100 °C 400 °C	4 min (100 °C) 1 hr (400 °C)	—	^54^
	WO_3_ NWs-Pt NPs	150 °C	1 hr	—	^55^
	SnO_2_ NWs-Cr_2_O_3_ NPs	700 °C	2 hr	Air	^56^
	ZnO NWs-CdS NPs	60 °C	40–250 min	—	^57^
	V_2_O_5_@ZnO-Au NPs	350 °C	1 hr	—	^58^
	ZnO NW-Au NPs	RT (25 °C)	12–18 hr	—	^59^
	SnO_2_ NFs-Pt NPs	150 °C	2 hr	—	^60^
	Zn_2_SnO_4_ NWs-ZnO QDs	95 °C	2 hr	—	^61^
	GaN NWs-TiO_2_ NCs	650–700 °C	30 s	—	^62^
	SnO_2_ NWs-NiO NPs	400 °C	5 hr	—	^63^
	SnO_2_ NWs-CdS QDs	60 °C 400 °C	30 min (60 °C) 2 hr (400 °C)	—	^64^
	ZnS NWs-CuO NPs	150 °C 500 °C	1 min (150 °C) 1 hr (500 °C)	1 mtorr	^65^
	TiO_2_ NWs-Ag NPs	50–60 °C	8 hr	—	^66^

As mentioned above, differences between science and engineering inevitably result in the need for a certain time period to achieve coincidence. In most cases, a theory is first established, and the corresponding time-saving potential is evaluated in the next step. Therefore, it is meaningful to find a new method allowing one to control several variables through a simple experiment, which can simplify the whole process but produce various results. In the meantime, we have invested a lot of time and money in the synthesis and functionalisation of new materials with novel properties. Thus, the know-how to produce the desired results with this simple method can be applied to other materials in the same way, and the search for even simpler and more powerful derivation methods should not be stopped.

## Conclusion

A new method of synthesising SnO_2_ and converting it to a different standard has been proposed. This method allows one to relatively easily control the parameters of Au-decorated SnO_2_ nanowires using thermal energy, i.e., the growth factors for each sample can be freely controlled depending on the given materials and processing time. Specifically, the reaction proceeds from a homogeneous structure to a heterogeneous structure, or from a stoichiometric structure to a non-stoichiometric structure, depending on the amount of applied energy. The trend of this transition is also consistent with the results of SEM, XRD, and TEM analyses. This seemingly ordinary process technology has proven to be overwhelmingly superior in terms of precursor, equipment, pre- and post-treatment, temperature, time, and vacuum. From an energy point of view, all experimentation with science/engineering bases relies on the idea of making a difference in energy or eliminating the energy difference. Thus, the ability to achieve a variety of effects by reducing the number of process variables and simply adjusting them should substantially contribute to reducing the congenital time gap between science and engineering.

## Methods

To prepare SnO_2_ nanostructures, Sn powder (1 g; Daejung Co., 99.9%) was placed in an alumina boat of a thermal evaporation furnace. The silicon substrate with 3-nm Au was placed upside down on the alumina boat to create conditions facilitating the adsorption of Au onto the substrate upon vaporisation. The temperature was raised to 900 °C at a rate of 10 °C/min, and an O_2_-Ar mixture (97:3) was flown at a pressure of 2 Torr for 1 h at 900 °C.

Gold Chloride hydrate 99.995% (HAuCl_4_·4H_2_O (0.23 g, 99.995%) and 2-propanol (10 g, 99.5%) were well mixed, and 3 mL of the mixture was dropped on the substrate part where the SnO_2_ nanowires were to be synthesised. Thereafter, a flame with a temperature of 1300 °C was applied for 5 s in the standby state using a special flame chemical vapour deposition (FCVD) technique.

Morphology was probed by scanning electron microscopy (SEM; Hitachi S-4200, Hitachi) and transmission electron microscopy (TEM; JEM-2100F, JEOL), crystallinity was probed by X-ray diffraction (XRD; Philips X’pert diffractometer, Philips) and high-resolution transmission electron microscopy (HRTEM), while elemental composition was probed by XRD, elemental mapping, and energy-dispersive X-ray spectroscopy (EDX).

## Supplementary information

Supplementary Information.

## Data Availability

All the data are available from the corresponding author on reasonable request.
