# Supplementary material for: Synthesis of Au/SnO2 nanostructures allowing process variable control
Source: Sci Rep. 2020 Jan 15;10:346. doi: 10.1038/s41598-019-57222-z (PMC6962171; doi:10.1038/s41598-019-57222-z)
Supplement: Supplementary file 1 — Supplementary Information. [file 41598_2019_57222_MOESM1_ESM.docx]

**Supplementary information**

**Synthesis of Au/SnO_2_ nanostructures allowing process variable control**

Myung Sik Choi^1^, Han Gil Na^1^, Sangwoo Kim^2^, Jae Hoon Bang^1^, Wansik Oum^1^, Sun-Woo Choi^3^, Sang Sub Kim^4^, Kyu Hyoung Lee^5^, Hyoun Woo Kim^1^* & Changhyun Jin^5^*

^1^Division of Materials Science and Engineering, Hanyang University, Seoul 04763, Republic of Korea

^2^Liquid Processing and Casting R&D Group, Korea Institute of Industrial Technology, 156, Getpearl-ro, Yeonsu-gu, Incheon 21999, Republic of Korea

^3^Department of Materials Science and Engineering, Kangwon National University, Samcheok, 25913, Republic of Korea

^4^Department of Materials Science and Engineering, Inha University, Incheon 402-751, Republic of Korea

^5^Department of Materials Science and Engineering, Yonsei University, Seoul, 03722, Republic of Korea

Myung Sik Choi, Han Gil Na, and Sangwoo Kim had equal contribution as co-first authors.

*Correspondence to: hyounwoo@hanyang.ac.kr (H. W. Kim), chjin0910@gmail.com (C. Jin)

**Table S1. Comparison of our process with previously reported ones.**

| **This work** | **Other work** | | | | |
| --- | --- | --- | --- | --- | --- |
| **Composites:**  SnO_2_ NWs - Au NPs  **Precursor:**  Gold Chloride hydrate,  2-propanol  **Equipment:**  FCVD equipment  **Pre- and post- treatment:**  Nothing | **Composites** | **Precursor** | **Equipment** | **Pre- and post- treatment** | **Ref.** |
|  | TiO_2_ NFs-Pt NPs | Ethylene glycol,  PVP,  H_2_PtCl_6_ | furnace | - | (S1) |
|  | TiO_2_ NFs-Pd NPs | Ethylene glycol,  PVP,  Na_2_PdCl_4_,  KBr,  H_2_O | furnace |  | (S1) |
|  | TiO_2_ NFs-Rh NPs | Ethylene glycol,  PVP,  Na_3_RhCl_6_ | furnace |  | (S1) |
|  | In_2_O_3_ NWs-TeO_2_ NPs | In_2_S_3_, Te | furnace | - | (S2) |
|  | SnO_2_ NWs-Pt NPs | MeCpPtMe_3_, O_2_, N_2_ | atomic layer deposition system | - | (S3) |
|  | ZnO NWs-Au NPs | HAuCl_4_·3H2O,  D.I water,  citrate trisodium salt | sonicator,  stirrer | sonication,  stirring | (S4) |
|  | ZnO NWs-Pt NPs | MeCpPtMe_3,_  O_2_, N_2_ | atomic layer deposition system | - | (S5) |
|  | WO_3_ NRs-Au NPs | PdCl_2_, ethanol, | UV irradiator, furnace | UV irradiation | (S6) |
|  | WO_3_ NRs-Pd NPs | HAuCl_4_, ethanol | UV irradiator, furnace | UV irradiation | (S6) |
|  | SiO_x_ NWs-Au NPs | Au | furnace | thin film deposition | (S7) |
|  | SiO_x_ NWs-Ag NPs | Ag | furnace | thin film deposition | (S7) |
|  | WO_3_ NWs-Co_3_O_4_ NPs | Co(CH_3_COO)_2_·4H_2_O, D.I water, NaOH, acetone, isopropyl alcohol | sonicator, centrifugal separator, furnace | sonication, centrifugation | (S8) |
|  | Si NWs-Pt NPs | H_2_PtCl_6_, ammonia | stirrer, furnace | stirring | (S9) |
|  | SnO_2_ NWs-Pd NPs | PdCl_2_, isopropanol, propanol | UV irradiator, furnace | UV irradiation | (S10) |
|  | SnO_2_@ZnO-Au NPs | HAuCl_4_·3.5H_2_O | γ-ray irradiator, furnace | γ-ray radiolysis | (S11) |
|  | In_2_O_3_ NRs-Bi_2_O_3_ NPs | Bi, In_2_S_3_ | furnace | - | (S12) |
|  | TiO NWs/NTs-Rh NPs | RhCl_3_·3H_2_O | furnace | - | (S13) |
|  | GaN NWs-SnO_2_ NPs | SnO_2_ target | RF sputter, furnace | sputtering | (S14) |
|  | ZnO NRs-Ag NPs | AgNO_3_, ethanol | stirrer,  UV irradiator, furnace | stirring,  UV irradiation | (S15) |
|  | TiO_2_ MRs-Ag NPs | AgNO_3_ | furnace | post-washing | (S16) |
|  | SiO_2_ NWs-Au NPs | Au | furnace | thin film deposition | (S17) |
|  | ZnO@TiO_2_@ZnSe-Ag NPs | Ag target | furnace,  DC sputter | sputtering | (S18) |
|  | SnO_2_ NWs-Pd NPs | PdCl_2_ | γ-ray irradiator, furnace | γ-ray radiolysis | (S19) |
|  | SnO_2_ NWs-Pt NPs | H_2_PtCl_6_·nH_2_O | γ-ray irradiator, furnace | γ-ray radiolysis | (S19) |
|  | SnO_2_ NWs-Au NPs | HAuCl_4_·nH_2_O | γ-ray irradiator, furnace | γ-ray radiolysis | (S19) |
|  | WO_3_ NRs-Pd NPs | PdCl_2_, NaCl, H_2_O | furnace, sonication, stirrer, centrifugal separator | pre-washing, sonication,  stirring, centrifugation | (S20) |
|  | WO_3_ NNs-Cu_2_O NPs | W(CO)_6_, Copper(II) acetylacetonate | furnace | - | (S21) |
|  | TiO_2_ NRs-CdSe NPs | Cadmium acetate dehydrate, sodium nitrilotriacetate, sodium selenosulfite | furnace | - | (S22) |
|  | ZnO NRs-Cds NPs | Cd target | pulsed laser deposition system | - | (S23) |
|  | ZnO NRs-Pd NPs | PdCl_2_, methanol, PVP | UV irradiator, stirrer | UV irradiation, stirring,  post-washing | (S24) |
|  | TiO_2_ NRs-Ag NPs | AgNO_3_, NaBH_4_, ethanol | - | post-washing | (S25) |
|  | ZnO NRs-Ag NPs | AgNO_3_, D.I water, ethanol | stirrer,  UV irradiator, vacuum oven | stirring,  UV irradiation, post-washing | (S26) |
|  | Fe­_2_O_3_ NRs-TiO_2_ NPs | Titanium tetra isopropoxide, isopropyl alcohol, NaOH, D.I water | sonicator, centrifugal separator, furnace | sonication, centrifugation | (S27) |
|  | WO_3_ NFs-Pd NPs | Ethylene glycol, PdCl_2_, PVP, acetone, ethanol | furnace, centrifugal separator | centrifugation, sonication, stirring | (S28) |
|  | WO_x_N_y_ NFs-Pt NPs | Ethylene glycol, chloroplatinic acid hexahydrate, PVP, acetone | furnace, centrifugal separator | centrifugation, decantation, sonication | (S29) |
|  | WO_3_ HTs-Pt NPs | Ethylene glycol, H_2_PtCl_6,_, PVP | furnace | - | (S30) |
|  | Cu_2_O NWs-Au NPs | NaBH_4_, HAuCl_4_, oleylamine, toluene, ethanol, chloroform | sonicator, stirrer, centrifugal separator | sonication,  stirring,  filtering, centrifugation | (S31) |
|  | CuO NWs-Au NPs | HAuCl_4_, D.I water, NaBH_4_, methanol | - | - | (S32) |
|  | Si NWs-Ag NPs | HF, AgNO_3_ | sonicator, centrifugal separator | sonication, centrifugation | (S33) |

**Table S2. Comparison of our process with previously reported ones.**

| **This work** | **Other work** | | | | |
| --- | --- | --- | --- | --- | --- |
| **Composites:**  SnO_2_ NWs - Au NPs  **Temp.:**  1300 ^o^C  **Time required:**  5 s  **Degree of vacuum:**  Air | **Composites** | **Temp.** | **Time required** | **Degree of vacuum** | **Ref.** |
|  | TiO_2_ NFs-  Pt NPs | 110 ^o^C  150 ^o^C | 1 hr (110 ^o^C)  3 hr (150 ^o^C) | - | (S1) |
|  | TiO_2_ NFs-  Pd NPs | 110 ^o^C  150 ^o^C | 1 hr (110 ^o^C)  3 hr (150 ^o^C) |  | (S1) |
|  | TiO_2_ NFs-Rh NPs | 110 ^o^C  150 ^o^C | 1 hr (110 ^o^C)  3 hr (150 ^o^C) |  | (S1) |
|  | In_2_O_3_ NWs-TeO_2_ NPs | 800 ^o^C | 1 hr | 1 torr | (S2) |
|  | SnO_2_ NWs-Pt NPs | 250 ^o^C | 65 min | - | (S3) |
|  | ZnO NWs-Pt NPs | 250 ^o^C | 65 min | - | (S5) |
|  | WO_3_ NRs-Pd NPs | 600 ^o^C | 1 hr | 1 torr | (S6) |
|  | WO_3_ NRs-Au NPs | 600 ^o^C | 1 hr | 1 torr | (S6) |
|  | SiO_x_ NWs-Au NPs | 900 ^o^C | 6 hr | - | (S7) |
|  | SiO_x_ NWs-Ag NPs | 900 ^o^C | 6 hr | - | (S7) |
|  | WO_3_ NWs-Co_3_O_4_ NPs | 150 ^o^C  500 ^o^C | 1 min (150 ^o^C)  1 hr (500 ^o^C) | - | (S8) |
|  | SnO_2_ NWs-Pd NPs | 500 ^o^C | 1 hr | - | (S10) |
|  | SnO_2_@ZnO-Au NPs | 500 ^o^C | 1 hr | - | (S11) |
|  | In_2_O_3_ NRs-Bi_2_O_3_ NPs | 800 ^o^C | 1 hr | 1 torr | (S12) |
|  | TiO NWs/NTs-Rh NPs | 656 ^o^C  746-1146 ^o^C | 3 hr (656 ^o^C)  1 hr (746-1146 ^o^C) | - | (S13) |
|  | GaN NWs-SnO_2_ NPs | 90 ^o^C  700 ^o^C | 7 min (90 ^o^C)  30 s (700 ^o^C) | - | (S14) |
|  | ZnO NRs-Ag NPs | 700 ^o^C | 1 hr | 1 torr- | (S15) |
|  | TiO_2_ MRs-Ag NPs | 160 ^o^C  250-700 ^o^C | 30 min (160 ^o^C)  2 hr (250-700 ^o^C) | - | (S16) |
|  | SiO_2_ NWs-Au NPs | 800 ^o^C | 6 min | - | (S17) |
|  | ZnO@TiO_2_@ZnSe-Ag NPs | 400 ^o^C | 2 min | - | (S18) |
|  | SnO_2_ NWs-Pd NPs | 500 ^o^C | 1hr | - | (S19) |
|  | SnO_2_ NWs-Pt NPs | 500 ^o^C | 1hr | - | (S19) |
|  | SnO_2_ NWs-Au NPs | 500 ^o^C | 1 hr | air | (S19) |
|  | WO_3_ NRs-Pd NPs | 45 ^o^C  400 ^o^C | 1 day (45 ^o^C)  2 hr (400 ^o^C) | - | (S20) |
|  | WO_3_ NNs-Cu_2_O NPs | 500 ^o^C | 3 hr | - | (S21) |
|  | TiO_2_ NRs-CdSe NPs | 350 ^o^C | 1 hr | - | (S22) |
|  | ZnO NRs-Cds NPs | 500 ^o^C | 10-30 min | - | (S23) |
|  | ZnO NRs-  Pd NPs | 25 ^o^C (RT) | 30 min | - | (S24) |
|  | TiO_2_ NRs-Ag NPs | 25 ^o^C (RT) | 25 s | - | (S25) |
|  | ZnO NRs-Ag NPs | 25 ^o^C (RT) | 10 min | - | (S26) |
|  | Fe­_2_O_3_ NRs-  TiO_2_ NPs | 150 ^o^C,  500 ^o^C | 1 min (150 ^o^C)  1 hr (500 ^o^C) | - | (S27) |
|  | TeO_2_ NRs-NiO NPs | 40 ^o^C  150 ^o^C  500 ^o^C | 1 hr (40 ^o^C)  1 min (150 ^o^C)  1 hr (500 ^o^C) | air | (S34) |
|  | TiO_2_ NRs-Cu NPs | 400 ^o^C | 1 hr | - | (S35) |
|  | In_2_O_3_ NRs-  Cr_2_O_3_ NPs | 500 ^o^C | 1 hr | air | (S36) |
|  | In_2_O_3_ NRs-  Co_3_O_4_ NPs | 160 ^o^C  500 ^o^C | 15 hr (160 ^o^C)  1 hr (500 ^o^C) | air | (S37) |
|  | WO_3_ NRs-  Cr_2_O_3_ NPs | 500 ^o^C | 1 hr | air | (S38) |
|  | SnO_2_ NWs-  PdO NPs | 400 ^o^C | 2 hr (400 ^o^C) | - | (S39) |

**References and Notes**

1. Formo, E., Yavuz, M.S., Lee, E.P., Lane, L. & Xia, Y. Functionalization of electrospun ceramic nanofibre membranes with noble-metal nanostructures for catalytic applications. *J. Mater. Chem. A* **19**, 3878-3882 (2009).
2. Park, S., Kheel, H., Sun, G.-J., Park, S.E. & Lee, C. Single-step synthesis of In_2_O_3_ nanowires decorated with TeO_2_ nanobeads and their acetone-sensing properties. *Appl. Phys. A-Mater*. **122**, 269 (2016).
3. Lin, Y.-H., *et al*. Fabrication of tin dioxide nanowires with ultrahigh as sensitivity by atomic layer deposition of platinum. *J. Mater. Chem*. **21**, 10552-10558 (2011).
4. Joshi, R.K., Hu, Q., Alvi, F., Joshi, N. & Kumar, A. Au decorated zinc oxide nanowires for CO sensing. *J. Phys. Chem. C* **113**, 16199-16202 (2009).
5. Lin, Y.-H., *et al*. Enhancing the photon-sensing properties of ZnO nanowires by atomic layer deposition of platinum. *ESC. Solid State Lett*. **13**, 93-95 (2010).
6. Kim, S., Park, S., Park, S. & Lee, C. Acetone sensing of Au and Pd-decorated WO_3_ nanorod sensors. *Sens. Actuators B-Chem*. **209**, 180-185 (2015).
7. Convertino, A., Cuscunà, M., Martelli, F., Manera, M.G. & Rella, R. Silica nanowires decorated with metal nanoparticles for refractive index sensors: Three-dimensional metal arrays and light trapping at plasmonic resonances. *J. Phys. Chem. C* **118**, 685-690 (2014).
8. Park, S., *et al*. Hydrogen gas sensing of Co3O4-decorated WO_3_ nanowires. *Met. Mater. Int*. **22**, 156-162 (2016).
9. Miao, F., Tao, B., Chu, P.K. Enhancement of the efficiency of dye-sensitized solar cells with highly ordered Pt-decorated nanostructured silicon nanowires based counter electrodes. *Electrochim. Acta* **96**, 61-65 (2013).
10. Kim, J.-H., Mirzaei, A., Kim, H.W., Kim, S.S. Improving the hydrogen sensing properties of SnO_2_ nanowire-based conductometric sensors by Pd-decoration. *Sens. Actuators B-Chem*. **285**, 358-367 (2019).
11. Kim, J.-H., Mirzaei, A., Kim, H.W., Kim, S.S. Low power-consumption CO gas sensors based on Au-functionalized SnO_2_-ZnO core-shell nanowires. *Sens. Actuators B-Chem*. **267**, 597-607.
12. Park, S., Kim, S., Sun, G.-J. & Lee, C. Synthesis, structure, and ethanol gas sensing properties of In_2_O_3_ nanorods decorated with Bi_2_O_3_ nanoparticles. *ACS. Appl. Mater. Interfaces* **7**, 8138-8146 (2015).
13. Pótári, G., *et al*. Rh-induced support transformation phenomena in titanate nanowire and nanotube catalysts. **29**, 3061-3072 (2013).
14. Bajpai, R., *et al*. UV-assisted alcohol sensing using SnO_2_ functionalized GaN nanowire devices. Sens. Actuators B-Chem. **171-172**, 499-507 (2012).
15. Park, S., An, S., Mun, Y., Kim, H.W. & Lee, C. Enhanced luminescence of Ag-decorated ZnO nanorod. *J. Mater. Sci.-Mater. Electron*. **24**, 4906-4912 (2013).
16. Amarjargal, A., Tijing, L.D., Pant, H.R., Park, C.-H. & Kim, C.S. Simultaneous synthesis of TiO_2_ microrods in situ decorated with Ag nanoparticles and their bactericidal efficiency. *Curr. Appl. Phys*. **12**, 1106-1112 (2012).
17. Colombelli, A., *et al*. Au nanoparticles decoration of silica nanowires for improved optical bio-sensing. *Sens. Actuators B-Chem*. **226**, 589-597 (2016).
18. Zhan, Li., *et al*. Optimized design of multi-shell ZnO/TiO_2_/ZnSe nanowires decorated with Ag nanoparticles for photocatalytic applications. *RSC. Adv*. **6**, 71800-71806 (2016).
19. Kim, J.-H., Wu, P., Kim, H.W. & Kim, S.S. Highly selective sensing of CO, C_6_H_6_, and C_7_H_8_ gases by catalytic functionalization with metal nanoparticles. *ACS. Appl. Mater. Interfaces* **8**, 7173-7183 (2016).
20. Tong, P.V., Hoa, N.D., Duy, N.V., Le, D.T.T. & Hieu, N.V. Enhancement of gas-sensing characteristics of hydrothermally synthesized WO_3_ nanorods by surface decoration with Pd nanoparticles. *Sens. Actuators B-Chem*. **223**, 453-460 (2016).
21. Annanouch, F.E., *et al*. Aerosol-assisted CVD-grown WO_3_ nanoneedles decorated with copper oxide nanoparticles for the selective and humidity-resilient detection of H_2_S. *ACS. Appl. Mater. Interfaces* **7**, 6842-6851 (2015).
22. Bang, J.H. & Kamt, P.V. Solar cells by design: Photoelectrochemistry of TiO_2_ nanorod arrays decorated with CdSe. *Adv. Funct. Mater*. **20**, 1970-1976 (2010).
23. Rakshit, T., Mondal, S.P., Manna, I. & Ray, S.K. CdS-decorated ZnO nanorod heterostructures for improved hybrid photovoltaic devices. *ACS. Appl. Mater. Interfaces* **4**, 6085-6095 (2012).
24. Chang, C.-M., Hon, M.-H. & Leu, I.-C. Outstanding H_2_ sensing performance of Pd nanoparticle-decorated ZnO nanorod arrays and the temperature-dependent sensing mechanisms. *ACS. Appl. Mater. Interfaces* **5**, 135-143 (2013).
25. Fang, H., Zhang, C.X., Liu, L., Zhao, Y.M. & Xu, H.J. Recyclable three-dimensional Ag nanoparticle-decorated TiO2 nanorod arrays for surface-enhanced Raman scattering. *Biosens. Bioelectron*. **64**, 434-441 (2015).
26. Chen, Y., Tse, W.H., Chen, L. & Zhang, J. Ag nanoparticles-decorated ZnO nanorod array on a mechanical flexible substrate with enhanced optical and antimicrobial properties. *Nanoscale Res. Lett*. **10**, 106 (2015).
27. Kheel, H., *et al*. Enhanced H_2_S sensing performance of TiO_2_-decorated α-Fe_2_O_3_ nanorod sensors. *Ceram. Int*. **42**, 18597-18604 (2016).
28. Kim, N.-H., *et al*. Highly sensitive and selective hydrogen sulfide and toluene sensors using Pd functionalized WO_3_ nanofibers for potential diagnosis of halitosis and lung cancer. *Sens. Actuators B-Chem*. **193**, 574-581 (2014).
29. Kim, D.-H., *et al*. Pt nanoparticles functionalized tungsten oxynitride hybrid chemiresistor: Low-temperature NO_2_ sensing. *Sens. Actuators B-Chem*. **273**, 1269-1277 (2018).
30. Choi, S.-J., *et al*. Selective diagnosis of diabetes using Pt-functionalized WO_3_ hemitube networks as a sensing layer of acetone in exhaled breath. *Anal. Chem*. **85**, 1792-1796 (2013).
31. Pan, Y., *et al*. Plasmon-enhanced photocatalytic properties of Cu_2_O nanowire−Au nanoparticle assemblies. *Langmuir* **28**, 12304-12310 (2012).
32. Mishra, A.K., *et al*. Superficial fabrication of gold nanoparticles modified CuO nanowires electrode for non-enzymatic glucose detection. *RSC Adv*. **9**, 1772-1781 (2019).
33. Peng, Z., *et al*. Heteroepitaxial decoration of Ag nanoparticles on Si nanowires: A case study on Raman scattering and mapping. *Nano Lett*. **10**, 3940-3947 (2010).
34. Park, S., Sun, G.-J., Khell, H., Choi, S. & Lee, C. Acetone gas sensing properties of NiO particle-decorated TeO_2_ nanorod sensors. *J. Nanosci. Nanotechnol*. **16**, 8589-8593 (2016).
35. Cheng, M., *et al*. Copper-decorated TiO_2_ nanorod thin films in optofluidic planar reactors for efficient photocatalytic reduction of CO_2_. *Int. J. Hydrog. Energy* **42**, 9722-9732 (2017).
36. Park, S., *et al*. Ethanol sensing properties of networked In_2_O_3_ nanorods decorated with Cr_2_O_3_-nanoparticles. *Ceram. Int*. **41**, 9823-9827 (2015).
37. Park, S., et al. Synergistic effects of codecoration of oxide nanoparticles on the gas sensing performance of In_2_O_3_nanorods. *Sens. Actuators B-Chem*. **227**, 591-599 (2016).
38. Choi, S., *et al*. Cr_2_O_3_ nanoparticle-functionalized WO_3_ nanorods for ethanol gas sensors. *Appl. Surf. Sci*. **432**, 241-249 (2018).
39. Lee, K.-C., Chiang, Y.-J., Lin, Y.-C. & Pan, F.-M. Effects of PdO decoration on the sensing behaviour of SnO­_2_ toward carbon monoxide. *Sens. Actuators B-Chem*. **226**, 457-464 (2016).
